# Dynamic causal modelling of precision and synaptic gain in visual perception — an EEG study

**DOI:** 10.1016/j.neuroimage.2012.06.044

**Published:** 2012-10-15

**Authors:** Harriet R. Brown, Karl J. Friston

**Affiliations:** Wellcome Trust Centre for Neuroimaging, UCL, London, UK

**Keywords:** Dynamic causal modelling (DCM), Electroencephalography (EEG), Gain, Precision, Effective connectivity, Visual contrast, Free Energy principle

## Abstract

Estimating the precision or uncertainty associated with sensory signals is an important part of perception. Based on a previous computational model, we tested the hypothesis that increasing visual contrast increased the precision encoded in early visual areas by the gain or excitability of superficial pyramidal cells. This hypothesis was investigated using electroencephalography and dynamic causal modelling (DCM); a biologically constrained modelling of the cortical processes underlying EEG activity. Source localisation identified the electromagnetic sources of visually evoked responses and DCM was used to characterise the coupling among these sources. Bayesian model selection was used to select the most likely connectivity pattern and contrast-dependent changes in connectivity. As predicted, the model with the highest evidence entailed increased superficial pyramidal cell gain in higher-contrast trials. As predicted theoretically, contrast-dependent increases were reduced at higher levels of the hierarchy. These results demonstrate that increased signal-to-noise ratio in sensory signals produce (or are represented by) increased superficial pyramidal cell gain, and that synaptic parameters encoding statistical properties like sensory precision can be quantified using EEG and dynamic causal modelling.

## Introduction

Predictive coding is an influential model of brain function that has proved helpful in explaining many visual phenomena, including extraclassical receptive field effects in V1 (Rao and Ballard, 1999), repetition suppression (Summerfield et al., 2008) and modulation of early cortical responses by attention (Rauss et al., 2011). Friston and Kiebel (2009) suggested a neurobiologically plausible scheme by which predictive coding could be executed in the cortex; key to these proposals is the idea that superficial pyramidal cells pass prediction error forward to higher cortical areas.

Classical predictive coding schemes are linear; however, these schemes cannot accommodate state dependent changes in the precision (inverse variability) of sensory signals. An important generalisation of predictive coding was introduced by Feldman and Friston (2010) to accommodate the fact that the precision of sensory signals is highly context sensitive and depends upon the (hidden) states of the world, generating sensory inputs. In the generalised predictive coding, precision scales the prediction error such that precise prediction errors have more influence at higher levels in representational cortical hierarchies; effectively, precision represents the signal-to-noise ratio or salience of prediction error associated with bottom-up signals. This sort of scaling is vitally important for sensory perception; for example, in multimodal integration (Ernst and Banks, 2002) and in reconciling information derived from sensory signals and prior knowledge (Rahnev et al., 2011a). Precision also influences the perception of visual contrast — increasing the relevance and the probability of a visual contrast signal have dissociable effects on energy sensitivity (Wyart et al., 2012), and attention allows the adoption of more stringent (conservative) detection criteria during contrast detection (Rahnev et al., 2011a, 2011b), suggesting that contrast detection is dependent on the estimated precision of sensory information. The introduction of precision also explains away the apparent contradiction between biased competition, which boosts expected signals (prediction errors) and predictive coding, which attenuates them (Feldman and Friston, 2010). This has recently been demonstrated experimentally; Kok et al. (2011) have shown, in an fMRI paradigm, that attention reverses the attenuation of BOLD signal seen in response to predictable stimuli. In generalised predictive coding, superficial pyramidal cells have been proposed to report precision-weighted prediction error, rather than pure prediction error (Friston and Kiebel, 2009). This sort of scheme is formally similar to those based upon adaptive resonance theory (Grossberg and Versace, 2008). See also Spratling (2008). Crucially, it provides a plausible mechanism for attentional modulation.

Recent theoretical work by the authors (Brown and Friston, 2012) has also investigated the role of precision in the context of visual illusions — in particular the Craik-O'Brien-Cornsweet (CBC) illusion. This illusion is perhaps the simplest of visual illusions, where a visual ‘edge’ between two isoluminant regions creates the impression that the one region is brighter than the other. We will focus on this illusion because it provides clear psychophysical evidence that contrast affects the encoding of precision in predictive coding — in a way that motivates the electrophysiological hypotheses tested in this paper. The CBC illusion was simulated by assuming observers make predictions about their visual input using a generative model in which reflectance and illuminance interact to produce sensory signals. Crucially, the generative model included prior beliefs that luminance varied with low spatial frequency whereas reflectance varied with high spatial frequency. Inversion of this generative model under a generalised predictive coding framework replicated the illusory perception of the CBC stimulus. In short, it was sufficient to explain the CBC illusion purely in terms of plausible prior beliefs about the spatial frequency structure of illuminance profiles and reflecting surfaces.

This is not the first explanation of the CBC to be based Helmhotlz's (Helmholtz, 1924) idea that the visual system must remove the effect of the illuminant to perceive. Purves et al. (1999, 2004) have suggested a Bayesian explanation for the CBC illusion and demonstrated that contextual cues indicating two differently reflectant surfaces subject to an illuminance gradient increase the magnitude of the CBC illusion. Mechanistically, this may be achieved by filling-in (Grossberg and Hong, 2006) — see also Reynolds and Heeger (2009) for a contemporary discussion in the context of the normalisation model.

**Fig. 1:**
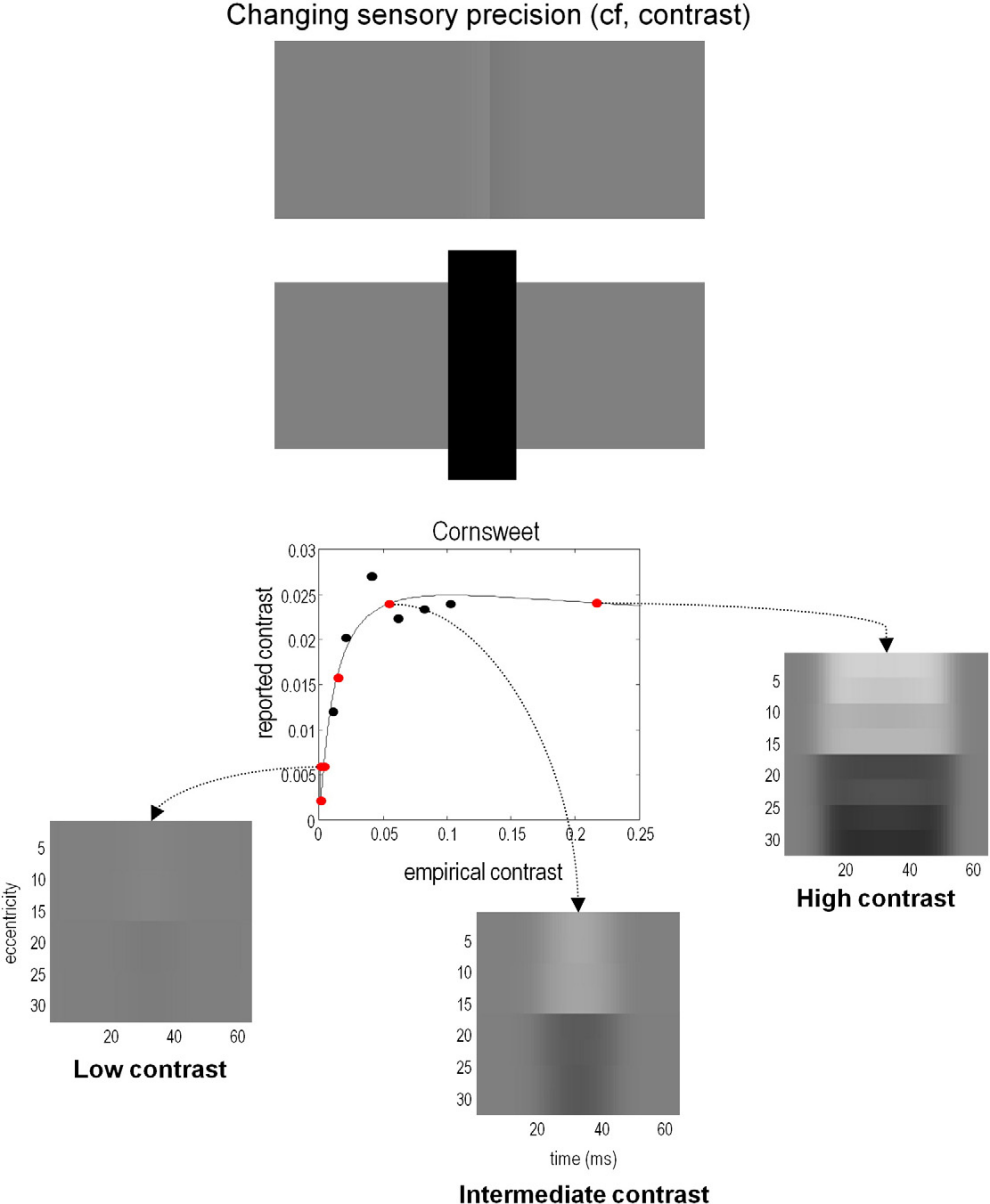
The Craik-O'Brien-Cornsweet (CBC) illusion. Upper panel: A demonstration of the CBC illusion. The two side panels have identical luminance. Close to the shared edge, there is a ramp of increasing luminance on the left and decreasing luminance on the right, which gives rise to the illusory percept that the panels have constant luminance and that there is a luminance step between them. Occluding the luminance ramps destroys this effect. Lower panel: psychophysical and simulated data from Brown and Friston (in submission). The black points are psychophysical data from a behavioural matching paradigm, in which the contrast of the stimulus luminance was varied and stimuli were matched to a real luminance step. The red points are simulated responses to the same stimuli, using a generalised predictive coding scheme; where the simulated and real psychophysical results have been scaled to match as closely as possible. Gamma values correspond to the log-precision of the (simulated) sensory input. Increasing the precision of sensory input reproduces the expression of the CBC illusion in human observers as visual contrast increases.

In these simulations, the emergence of the CBC illusion depends critically on the luminance contrast of the stimuli. To simulate the effects of changing contrast, the precision of the sensory inputs (the first hierarchical layer of the generative model) was manipulated; high precision corresponded to higher contrast and vice versa. This manipulation was based on Weber's law (Weber, 1846) and evidence that the brain uses divisive normalisation (Brady and Field, 2000; Carandini and Heeger, 1994), meaning that higher contrast stimuli have a higher signal-to-noise ratio and therefore higher precision. Associating stimulus contrast with precision enabled the generalised predictive coding scheme to accurately reproduce the effects of contrast on human observers; namely, the magnitude of the CBC illusion increased to a plateau (Fig. 1), and Mach bands appeared at higher contrast. These results suggest that increasing stimulus contrast increases the precision of sensory signals encoded by early visual areas.

In the context of the generalised predictive coding scheme outlined above, precision is encoded by the gain of superficial pyramidal cells. Gain in early visual areas is known to be important for contrast perception. Cells in visual area V1 are sensitive to contrast and, on a timescale that precludes neuronal adaptation, the firing rate of such cells generally increases linearly in response to increasing visual contrast, except at very high contrast levels (Albrecht et al., 1984; Ohzawa et al., 1982). Although this pattern is consistent with a gain-based explanation of visual contrast coding, this is by no means the only explanation. In this study, we used EEG and dynamic causal modelling to investigate role of synaptic gain in contrast perception in early and higher cortical areas.

## Materials and methods

### Participants

18 healthy right-handed subjects participated in the study (9 males; aged 20–56). Ethical approval was obtained from the UCL Research Ethics Committee (no. 2715/002). Written informed consent was obtained from all subjects.

### Experimental paradigm

CBC stimuli (Fig. 7) were created by applying a bandpass filter to 1024 × 512 arrays of white noise to produce a random blob pattern with a fundamental frequency of 67 blobs/image (1 cycle/degree). This pattern was thresholded and convolved with a 2-D Laplacian-of-Gaussian filter to give a CBC stimulus. Stimuli were scaled to have 10%, 25% or 90% of the maximum contrast supported by the monitor. The stimuli occupied both lower quadrants of the screen, subtending approximately 32° of visual angle. The central 2° of visual angle were left blank. Stimuli were presented against a grey background on a gamma-corrected monitor. Average luminance was 48 cd/m^2^.

Participants sat on a comfortable chair and rested their head on a chin rest. The stimuli were displayed on an LCD monitor 60 cm from the subjects. During the task, subjects fixated on a central cross. One of the three CBC stimuli was presented on the bottom half of the screen for 400 ms. Inter-trial interval was jittered between 600 ms and 800 ms. Three sessions of 1200 stimuli were presented, over about one hour's scanning time. During the task, the fixation cross changed to a circle and back again between stimuli, randomly with a probability of 0.01, to provide targets for an incidental task, used to maintain attentional set. Participants counted these events and reported the total to the experimenter after each session.

### Data collection and processing

EEG data were recorded using a *Biosemi* system with 128 scalp electrodes at a sampling rate of 512 Hz. An average reference was used. Vertical and horizontal eye movements were monitored with electro-oculogram electrodes. Electrode positions were recorded with a Polhemus digitiser. Data were analysed using SPM8 (http://www.fil.ion.ucl.ac.uk/spm/software/spm8/).

Data were down-sampled to 200 Hz and bandpass-filtered between 0.5 Hz and 45 Hz to suppress very low frequencies. Baseline-corrected epochs were extracted from the time series starting at 100 ms before stimulus onset and ending at 400 ms after stimulus onset. Blink and eye-movement artefacts were detected by simple thresholding of electro-oculogram channels; artefactual trials were removed from the analysis. 9.7% of trials were excluded (range across subjects 0.3%–32%). Three types of event related averages were taken — an average for each subject and contrast level, an average over contrast levels for each subject and an average for each contrast level over all subjects.

### Source localisation

**Fig. 2:**
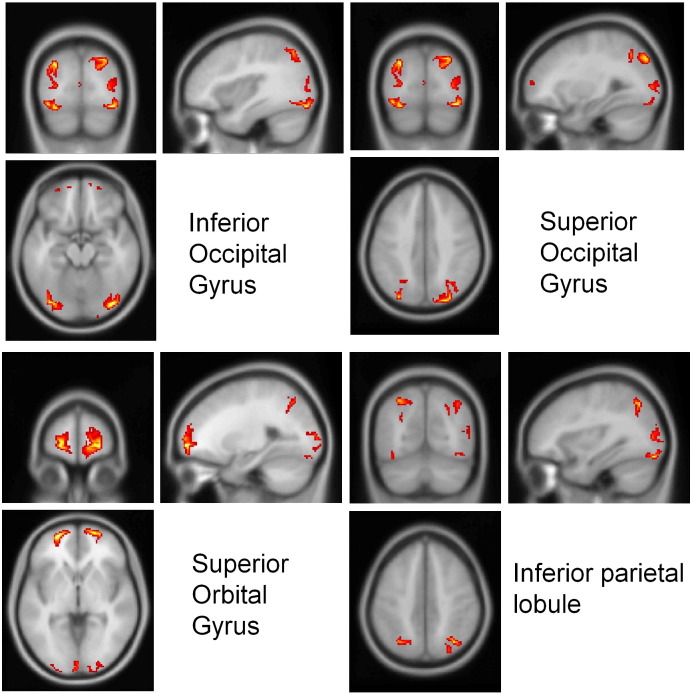
Source localisation. Sources located by source reconstruction using multiple sparse priors and group constraints. The figures show absolute source activity averaged across subjects; the maxima were used as source locations for DCM. Four locations emerged bilaterally: the inferior occipital gyrus, the inferior parietal cortex, the superior occipital gyrus and the superior orbital gyrus.

Using the event related potentials averaged over contrasts for each subject, source localisation was performed using multiple sparse priors and group constraints (Litvak and Friston, 2008). This localisation optimises prior covariance constraints on sources over subjects and provides maximum a posteriori estimates of activity at each source from a cortical mesh from 60 ms post stimulus onset to 400 ms post stimulus onset for each subject. These estimates were averaged over peristimulus time and projected to a three-dimensional source space, where they were smoothed to create an image of source activity for each subject. Individual subject images were averaged. This procedure was used to identify the location of four bilateral sources in each hemisphere (see Fig. 2). The sources were identified as the four bilateral peaks with the largest posterior estimates of evoked power (sum of squared source activity over peristimulus time — Litvak and Friston, 2008).

### DCM

We used dynamic causal modelling as implemented by SPM8 to examine the changes in pyramidal cell gain due to changes in visual contrast (Kiebel et al., 2008). Dynamic causal modelling employs biophysically constrained models and a Bayesian inversion scheme to infer hidden variables relating to connectivity and synaptic efficacy by modelling EEG data as the response of a dynamic input-state-output system to experimental perturbations. The model comprises both a neuronal mass model that allows for directed coupling among hidden neuronal states and the electromagnetic forward model (used for source localisation above) that maps from hidden neuronal states to observed channel data.

The neuronal model employed in DCM consists of a number of discrete cortical sources, each comprising four cell populations — superficial and deep pyramidal cells, spiny stellate cells and inhibitory interneurons. The activity of these populations is coupled by a series of differential equations, which are based on the intrinsic connectivity among cortical layers (Bastos et al., 2011). A series of parameters, (*γ*_1_–*γ*_10_) specifies the strength of intrinsic connectivity between populations; four of the intrinsic connections are optimised to fit the data, the others are fixed. One or more may be optimised in a condition-specific way.

Extrinsic connections link different sources. Extrinsic forward connections are excitatory, originate from superficial pyramidal cells and terminate on spiny stellate neurons. Extrinsic backward connections are inhibitory, originate from deep pyramidal cells and terminate on superficial pyramidal cells. Under generalised predictive coding, superficial pyramidal cells are thought to signal precision-weighted prediction error (Friston and Kiebel, 2009). The precision is represented in the model by pyramidal cell self-connectivity, *γ*_*7*_.

To generate predicted signals in sensor space, superficial pyramidal cell activity (which is thought to represent most of the EEG signal) is multiplied by a lead field matrix which maps sources to sensors to produce simulated data. This lead field matrix constitutes the conventional electromagnetic forward model.

The dynamic causal model is inverted using variational Bayesian procedures to obtain the posterior density of the free parameters given the data. As well as the four intrinsic connection strength parameters, our free parameters included the strength of all extrinsic connections. The posterior distributions were obtained using a standard Variational Laplace scheme as described in Friston et al. (2007).

To determine the connectivity of the areas identified by the source localisation, Bayesian model selection was first performed using the free energy, which is an approximation to log model evidence. Six plausible models were specified (Fig. 8), representing both parallel and serial hierarchies, with and without inter-hemisphere connections. Each of the six models was fitted separately to the average response over all subjects for each contrast level. A fixed-effects model comparison was then performed.

The winning model was used for all subsequent analyses. Within this model, three sub-models of contrast-dependent effects were evaluated using subject-specific averages: a model with no contrast-dependent effects, a model with contrast-dependent changes in the self-connectivity of superficial pyramidal cells (*γ*_7_) and a model allowing contrast-dependent changes in the self-connectivity of deep pyramidal cells (*γ*_10_). A fixed effects Bayesian model comparison was then used to compare the final three models (contrast dependent effects upon the superficial, deep or no cells) by pooling their log evidences over subjects.

### Statistical analysis

Statistical analysis of the parameter estimates from the winning model was performed in SPSS 20.0. The winning model had contrast-dependent changes in the γ_7_ parameter (self‐inhibition of superficial pyramidal cells or negative gain). The maximum *a posteriori* estimates of the changes in these parameters were quantified using a classical summary statistic approach. Eight parameters changed in a contrast-specific way in each subject-specific model — one for each of four areas in both hemispheres. These parameters were entered into a two-way ANOVA with factors cortical source (with four levels) and hemisphere (with two levels). In addition, a one-way ANOVA with planned contrast testing for a (linear) change in gain with hierarchical level was performed, weighting the groups (from the bottom of the hierarchy to the top) as 4,3,2,1. Equal variance was assumed.

## Results

Source localisation revealed four bilateral sources of activity (Fig. 2): inferior occipital (IOG), the inferior parietal cortex (IPC), superior occipital gyrus (SOG) and the superior orbital gyrus (SOrbG). These cortical areas have been implicated previously in the processing of visual form and the global (spatial) attributes of visual stimuli (Peterson et al., 1999; Podzebenko et al., 2005; Shikata et al., 2003). The locations of these sources were used in subsequent dynamic causal modelling of observed responses in sensor space. Note that our anatomical designations are just mnemonic. Although our source reconstruction used a canonical template — and the sources can be associated with a Talairach and Tournoux location — the spatial precision of EEG source reconstruction means that anatomical localisation is very approximate.

**Fig. 3:**
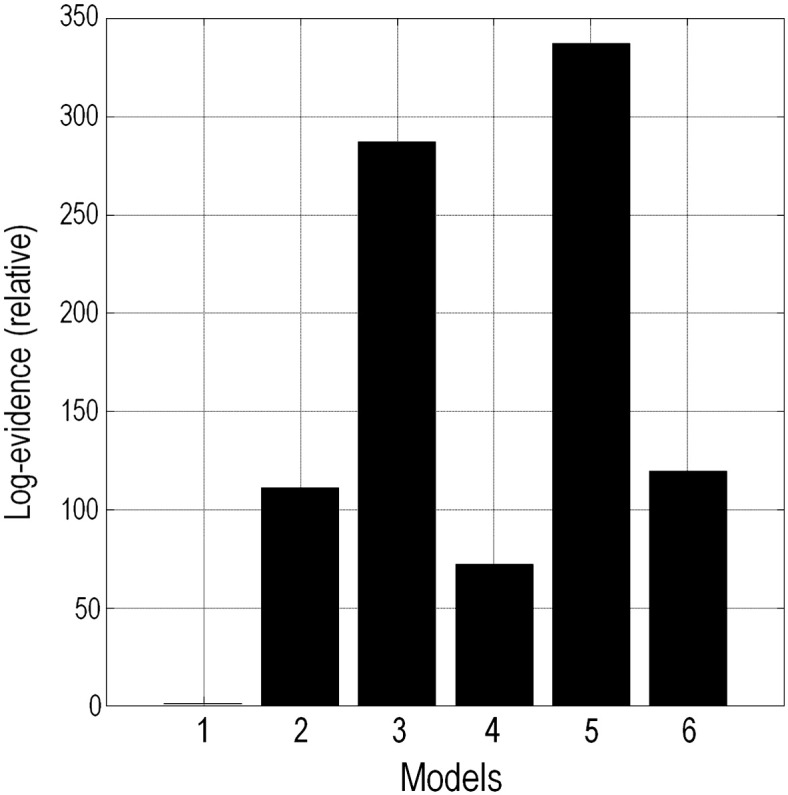
Results of fixed-effects Bayesian Model Selection. Upper panel: out of the different extrinsic connectivity models, Model 5, a serial hierarchy with interhemispheric connections, had the most evidence. This model was used for subsequent analyses.

**Fig. 4:**
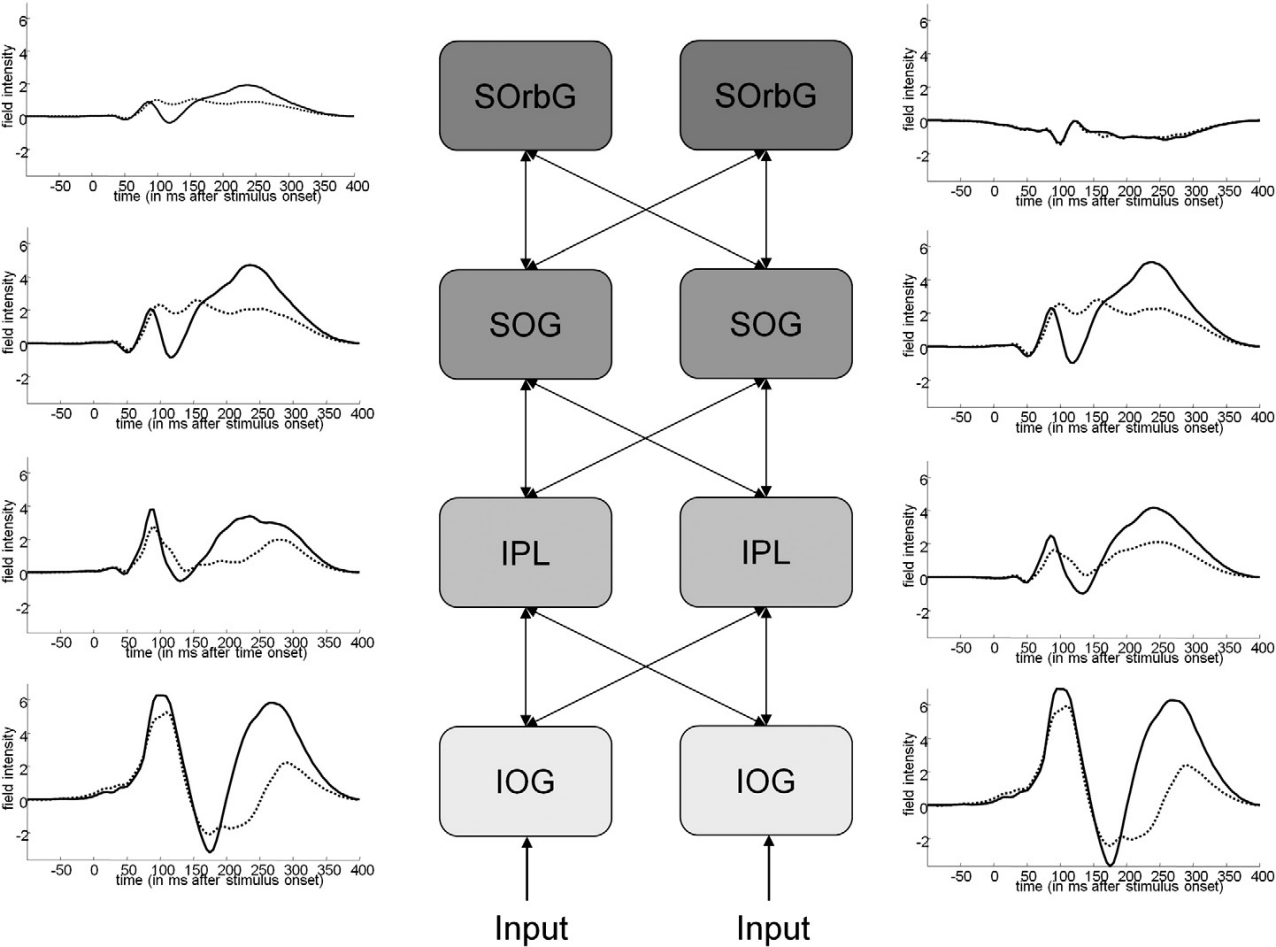
Prediction error in the cortical hierarchy. This figure shows the activity reconstructed at each of the sources used for DCM analysis (based on a DCM of the grand average event related potentials over subjects). These responses can be taken to be a rough proxy for prediction error, since superficial pyramidal cells contribute most of the EEG signal. The difference in signal between high-contrast and low-contrast clearly reduces as the hierarchy is ascended, reflecting the decreasing differences in the precision of prediction error.

Six dynamic causal models (Kiebel et al., 2008) employing a canonical microcircuit model of neural activity (Bastos et al., 2011) were fitted to the event related potentials averaged over all subjects for each level of contrast (Fig. 8). Fixed effects Bayesian model comparison was used to compare the evidence for each model, pooled over subjects. The model with the greatest evidence was a simple hierarchy with diagonal interhemispheric connections (Fig. 3, Fig. 4). This model was then used to assess contrast-dependent changes in coupling for each subject.

**Fig. 5:**
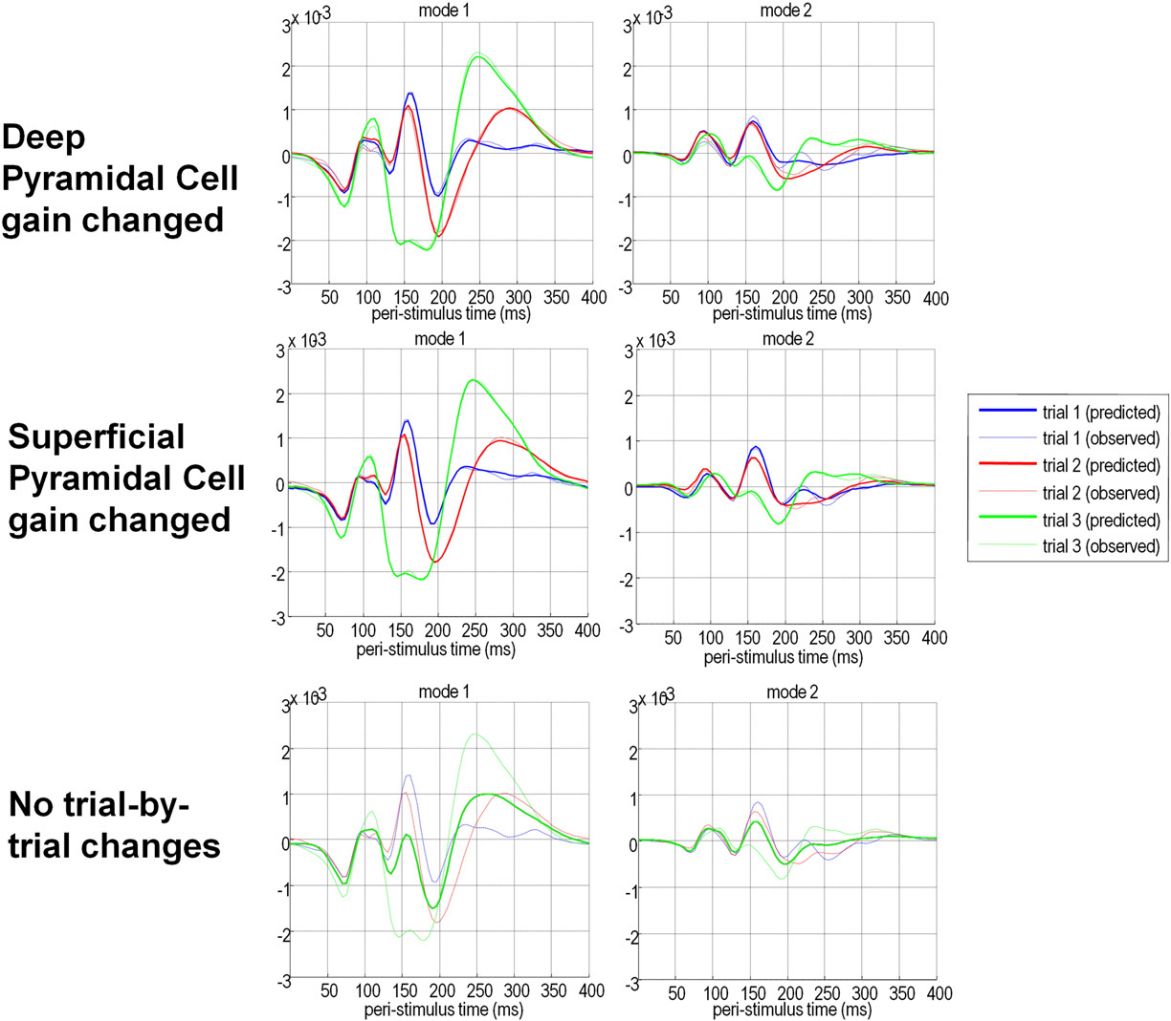
Model fits. The fits of the three models of contrast-dependent effects to event related potentials in sensor-space for an illustrative subject; these responses are summarised with the first two principal components or modes. The modes are used for data reduction — the data are projected onto the principal eigenvectors of the prior covariance of the data. In this paper, eight modes are used in total. The dashed lines show the data modes and the solid lines the model predictions. In the best-fitting model (centre) these are almost superimposed, whereas in the less well-fitting models, substantial differences are evident.

This model was fitted to individual subject data with three possible models of contrast-dependent effects; one which allowed no changes in connectivity, a model with contrast-dependent changes in the self-connectivity of superficial pyramidal cells and a model allowing contrast-dependent changes in the self-connectivity of deep pyramidal cells. Fixed-effects Bayesian model comparison showed the model with contrast-dependent changes in the self-connectivity (gain) of the superficial pyramidal cells had the most evidence, with a log Bayes factor of 900 — compared to equivalent model with changes in the deep pyramidal cells. Both of these models had an overwhelming amount of evidence in relation to the null model, with no contrast dependent changes in gain (with Bayes factors of over 30,000). The model with contrast dependent changes in superficial pyramidal cell gain provided an excellent fit to the data (Fig. 5).

**Fig. 6:**
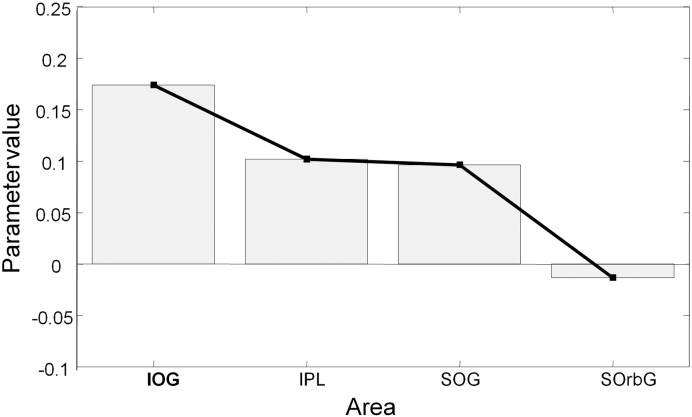
Contrast-dependent changes in the game of superficial pyramidal cells. These are the average parameters, over subjects, controlling the contrast dependent changes in negative self-inhibition (gain) under the winning model of the previous figures. Note the progressive decrease in contrast-dependent effects at higher levels of the hierarchy. This is predicted theoretically, because we have manipulated the precision of prediction errors at the lowest (sensory level) through experimental manipulations of visual contrast.

**Fig. 7:**
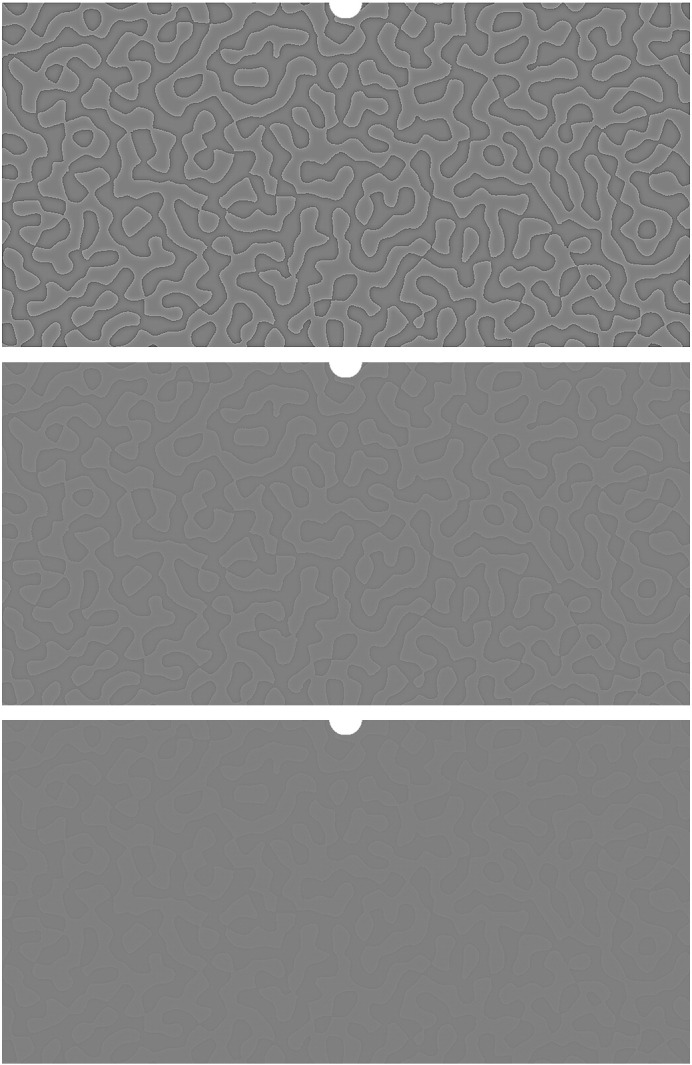
CBC stimuli used for this study. These stimuli were created by applying a bandpass filter to white noise to create a random blob pattern with a fundamental frequency of 67 blobs/image (1 cycle/degree). This pattern was thresholded and convolved with a 2-D Laplacian-of-Gaussian filter to produce a CBC stimulus. Stimuli were scaled to have 90% (top), 25% (middle) or 10% (bottom) of the maximum contrast supported by the monitor. The stimuli subtended approximately 32° of visual angle. The central 2° of visual angle were left blank. Stimuli were presented against a grey background on a gamma-corrected monitor.

**Fig. 8:**
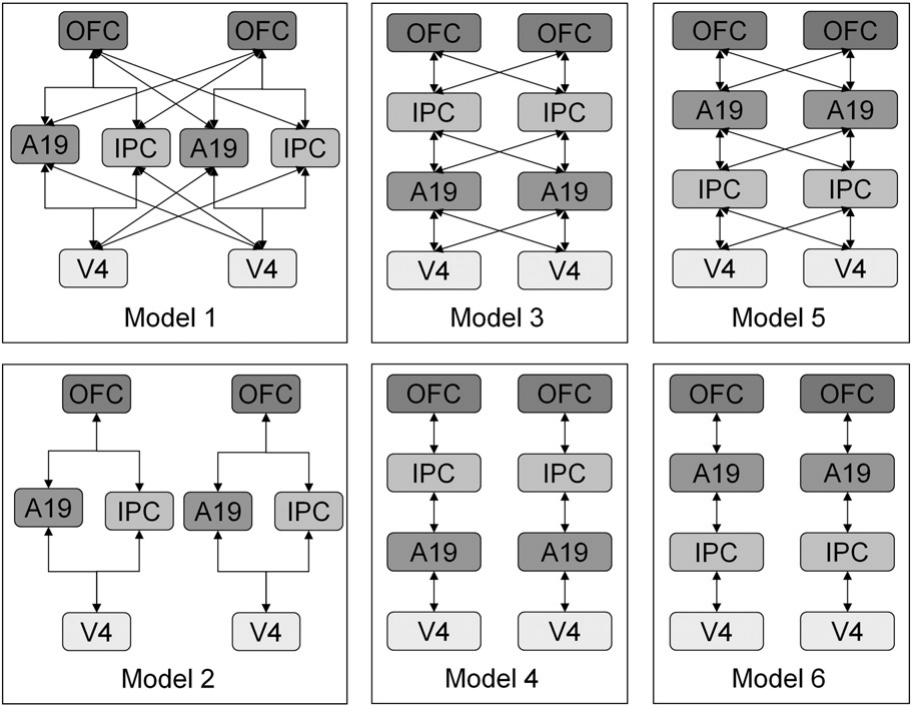
Model selection. Initial model selection was carried out to identify the extrinsic connectivity pattern on the sources identified. Only plausible models were tested; these models had the inferior orbital gyrus at the bottom of the hierarchy and the superior orbital gyrus at the top (Felleman and Van Essen, 1991). These models are distinguished by the deployment of forward and backward extrinsic connections, as determined by their level in the hierarchy. Here, this level corresponds to vertical position.

Contrast-dependent changes in coupling under the winning model were assessed in a *post hoc* fashion, using classical inference. Two-way ANOVA (with factors cortical source and hemisphere) showed no effect of side (*F*_1,136_ = 1.850; *p* = 0.073), so parameters pertaining to left and right sources were analysed together subsequently. One-way ANOVA with planned testing for a (linear) change in gain with hierarchical level showed a significant trend for contrast-dependent increases in lower sources and smaller, or no, contrast-dependent increases in higher sources of the hierarchy (*t*_140_ = − 2.472; *p* = 0.015) (Fig. 6). The contrast-dependent changes in gain shown in Fig. 6 produce a progressive attenuation of contrast-dependent effects at higher levels in the hierarchy. This can be seen easily in Fig. 4, where solid lines represent the highest-contrast condition and dotted lines the lowest-contrast condition. The difference in responses to the different levels of contrast clearly decreases as the hierarchy is ascended.

## Discussion

The results of this study suggest that the visual contrast of a stimulus increases the gain of superficial pyramidal cells in lower visual areas, relative to higher levels. This is entirely consistent with generalised predictive coding, where visual contrast determines the precision of sensory signals and the representation of that precision in terms of the gain or sensitivity of superficial pyramidal cells.

Generalised predictive coding suggests that forward connections in the brain (known to originate from superficial pyramidal cells (Felleman and van Essen, 1991; Maunsell and van Essen, 1983) convey precision-weighted prediction error. Theoretical work described above (Brown & Friston, in submission) has shown that increasing visual contrast corresponds to increasing the precision of sensory channels in accordance with Weber's law. In this study, we have shown that the changes in the EEG signal across levels of visual contrast can be modelled by changes in gain in superficial pyramidal cells. This gain is thought to represent the precision of the prediction error, which determines the signal-to-noise ratio associated with sensory input.

A technical issue — that deserves a brief comment — is that the cell populations, whose intrinsic gain best models visual contrast effects, are the same populations generating the observed EEG signal (in the model). One might ask whether this biases our model comparison, given that visually evoked responses generally increase with contrast (Polat and Norcia, 1996). Although this is a possibility — in the sense that any inference in DCM pertains only to the models considered — the intrinsic connections between superficial and deep cells means that changes in the gain of deep cells could also easily explain the contrast dependent responses — through their influence on superficial cells. Furthermore, models with contrast dependent changes in extrinsic connections (targeting both superficial and deep populations) had substantially lower evidence than the models reported above (results not shown). In short, an increase in the gain superficial pyramidal cells appears to be the best explanation for contrast dependent effects, within the alternative models that we could conceive of.

It should be noted, that a contrast dependent increases in evoked responses could be modelled in many ways. In this sense, our use of DCM can be regarded as testing specific hypotheses about a limited number of competing explanations. We focused on the gain or intrinsic sensitivity of superficial and deep pyramidal cells because explanations in terms of post-synaptic gain follow directly from predictive coding formulations of perceptual synthesis. This does not mean that other hypotheses could be explored based upon alternative theoretical formulations. In brief, as with all dynamic causal modelling studies, our conclusions have to be qualified in relation to the hypotheses all models addressed.

On a general note, the conclusions of this paper highlight the utility of dynamic causal modelling — in using experimental data to ask specific questions. Fig. 5 shows that the effects of changing the gain of superficial and deep pyramidal cells are, qualitatively, very similar. This means that we are faced with a very difficult problem in adjudicating between implicit explanations for contrast dependent effects. Note that this problem cannot be finessed experimentally — for example, there is no (non-invasive) experimental manipulation of contrast that selectively engages deep or superficial pyramidal cells. The solution offered by DCM is to place constraints on the way that data are explained and use Bayesian modelling to quantify the evidence for different hypotheses. Note that although the expression of the different hypotheses in Fig. 5 looks very similar, the evidence for contrast dependent changes in the gain of superficial cells is enormous (with a Bayes factor of over 900). This evidence could not be intuited by simply looking at the data: it is disclosed by careful and informed Bayesian modelling of those data. In short, dynamic causal modelling of this sort exploits prior knowledge to solve otherwise very difficult inference problems. However, this solution rests upon the specification of specific and well posed questions. In other words, the efficiency with which this sort of modelling adjudicates between different hypotheses depends on an efficient and careful experimental design.

The cells of early visual areas corresponding to human V1 have been studied extensively with electrophysiologically in cat and macaque. Although our study did not model V1 as a distinct source, the lack of spatial resolution with EEG means that the results from IOG can be regarded as representative of early visual responses. The supragranular superficial pyramidal cells in our dynamic causal model are located in the same cortical layers as complex cells in cat area 17, which predominate in layers 2 and 3 (Gilbert and Wiesel, 1979). Moreover, in Rao and Ballard's predictive coding model of visual cortex, prediction error units display complex cell-like behaviour in the presence and absence of feedback (Rao and Ballard, 1999). Subsequent examination of the contrast-dependent responses of such cells shows that, in the absence of adaptation, their firing rate generally increases linearly in response to increasing visual contrast, except at very high contrast levels (Albrecht et al., 1984; Ohzawa et al., 1982). The short stimulus duration and dim screen used in our study suggests we can discount adaptation of retinal or early cortical responses and therefore contrast-dependent responses at the cellular level should increase monotonically with contrast, which seems to be the case in Fig. 4. Dynamic causal modelling of the underlying synaptic mechanisms suggests that this increase is the result of increasing gain in superficial pyramidal cells.

What might be the mechanism behind these gain increases? In perceptual processing, acetylcholine signalling seems to be an important mechanism for contrast gain-control. Increasing endogenous acetylcholine reduces contrast-dependent gain (DeBruyn et al., 1986), while nicotine has a suppressive effect on gain in cortical layers 2,3 and 5 but an facilitatory effect in layer 4c, where stellate cell bodies are located (Disney et al., 2007). Short-term depression, particularly at the thalamocortical synapse to spiny stellate cells, has also been proposed to play a role (Carandini et al., 2002; Chung et al., 2002); this would fit with the neuronal encoding of precision by neuromodulatory mechanisms.

The mechanisms discussed in relation to encoding precision also seem to have an important role in contrast sensitivity. For example, nicotine increases contrast sensitivity (Disney et al., 2007), especially at low spatial frequencies (Smith and Baker-Short, 1993), while scopolamine, a muscarinic antagonist, universally increases contrast sensitivity (Smith & Baker-Short, 1993), an effect that can be attenuated by increasing the luminance (precision) of the stimulus (Evans, 1975).

Aberrant encoding of precision and uncertainty has been proposed to play a role in a number of neuropsychiatric disorders. In patients with schizophrenia, the mismatch negativity, an evoked potential that is greater in response to deviant or unexpected auditory tones, is decreased in magnitude (Jahshan et al., 2011; Jordanov et al., 2011; Leitman et al., 2010). This may represent a failure to detect statistical regularities and assign higher precision to sensory information, leading to a reduced difference between responses to standard and deviant stimuli (Garrido et al., 2008; Garrido et al., 2009; Strelnikov, 2007). In other words, schizophrenic subjects may never be surprised because they fail to make precise predictions. This explanation for the mismatch negativity speaks to an optimisation of precision or gain associated with sensory prediction errors due to rapid sensory learning and calls upon exactly the same synaptic mechanisms that have been proposed to at mediate attentional gain (Feldman and Friston 2010). In short, sensory surprise depends upon appropriately precise prediction errors and adaptive precision or gain control.

Corlett et al. (2009) have proposed a hierarchical Bayesian explanation for schizophrenia that rests on the aberrant weighting of top-down and bottom-up information that could lead to both hallucinations and delusions. In predictive coding, this weighting is determined by the precision of prediction errors at different levels in hierarchical generative models. Patients with schizophrenia show a pan-frequency increase in contrast sensitivity threshold (Skottun and Skoyles, 2007; Slaghuis, 1998), which could reflect inadequate increase of synaptic gain at superficial pyramidal cells in response to high-contrast stimuli. These ideas are important, because our study suggests it is possible to measure the neuronal encoding of precision noninvasively using EEG, in a very simple paradigm which would be easy to perform with patients.

In conclusion, we have provided evidence that the contrast-dependency of early visual cortical responses is mediated by the gain of superficial pyramidal cells. In computational terms, this gain may encode the precision of prediction errors signalled by these cells. These results suggest that DCM may be useful as an assay of the synaptic (neuromodulatory) mechanisms that underlie perceptual inference.

## References

[bb0005] Albrecht D.G., Farrar S.B., Hamilton D.B. (1984). Spatial contrast adaptation characteristics of neurones recorded in the cat's visual cortex. J. Physiol..

[bb9050] Bastos A., Moran R., Litvak V., Fries P., Friston K. (2011).

[bb0010] Brady N., Field D.J. (2000). Local contrast in natural images: normalisation and coding efficiency. Perception.

[bb9055] Brown H., Friston K.J. (2012). Free-energy and illusions: the Cornsweet effect. Front. Psychol..

[bb0015] Carandini M., Heeger D.J. (1994). Summation and division by neurons in primate visual cortex. Science.

[bb0020] Carandini M., Heeger D.J., Senn W. (2002). A synaptic explanation of suppression in visual cortex. J. Neurosci..

[bb0025] Chung S., Li X., Nelson S.B. (2002). Short-term depression at thalamocortical synapses contributes to rapid adaptation of cortical sensory responses in vivo. Neuron.

[bb0035] Corlett P.R., Frith C.D., Fletcher P.C. (2009). From drugs to deprivation: a Bayesian framework for understanding models of psychosis. Psychopharmacology.

[bb0040] DeBruyn E.J., Gajewski Y.A., Bonds A.B. (1986). Anticholinesterase agents affect contrast gain of the cat cortical visual evoked potential. Neurosci. Lett..

[bb0045] Disney A.A., Aoki C., Hawken M.J. (2007). Gain modulation by nicotine in macaque v1. Neuron.

[bb0050] Ernst M.O., Banks M.S. (2002). Humans integrate visual and haptic information in a statistically optimal fashion. Nature.

[bb0055] Evans H.L. (1975). Scopolamine effects on visual discrimination: modifications related to stimulus control. J. Pharmacol. Exp. Ther..

[bb0060] Feldman H., Friston K.J. (2010). Attention, uncertainty, and free-energy. Front. Hum. Neurosci..

[bb0065] Felleman D.J., Van Essen D.C. (1991). Distributed hierarchical processing in the primate cerebral cortex. Cereb. Cortex.

[bb0070] Friston K., Kiebel S. (2009). Predictive coding under the free-energy principle. Philos. Trans. R. Soc. Lond. B Biol. Sci..

[bb9000] Friston K., Mattout J., Trujillo-Barreto N., Ashburner J., Penny W. (2007). Variational free energy and the Laplace approximation. NeuroImage.

[bb9005] Garrido M.I., Friston K.J., Kiebel S.J., Stephan K.E., Baldeweg T., Kilner J.M. (2008). The functional anatomy of the MMN: a DCM study of the roving paradigm. NeuroImage.

[bb9010] Garrido M.I., Kilner J.M., Kiebel S.J., Friston K.J. (2009). Dynamic causal modeling of the response to frequency deviants. Journal of Neurophysiology.

[bb0075] Gilbert C.D., Wiesel T.N. (1979). Morphology and intracortical projections of functionally characterised neurones in the cat visual cortex. Nature.

[bb9015] Grossberg S., Hong S. (2006). A neural model of surface perception: lightness, anchoring, and filling-in. Spat. Vis..

[bb0080] Grossberg S., Versace M. (2008). Spikes, synchrony, and attentive learning by laminar thalamocortical circuits. Brain Res..

[bb9020] Helmholtz H. von (1924).

[bb9025] Jahshan C., Cadenhead K.S., Rissling A.J., Kirihara K., Braff D.L., Light G.A. (2011). Automatic sensory information processing abnormalities across the illness course of schizophrenia. Psychol. Med..

[bb9030] Jordanov T., Popov T., Weisz N., Elbert T., Paul-Jordanov I., Rockstroh B. (2011). Reduced mismatch negativity and increased variability of brain activity in schizophrenia. Clin. Neurophysiol..

[bb0085] Kiebel S.J., Garrido M.I., Moran R.J., Friston K.J. (2008). Dynamic causal modelling for EEG and MEG. Cogn. Neurodyn..

[bb0090] Kok P., Rahnev D., Jehee J.F.M., Lau H.C., de Lange F.P. (2011). Attention reverses the effect of prediction in silencing sensory signals. Cereb. Cortex.

[bb9040] Leitman D.I., Sehatpour P., Higgins B.A., Foxe J.J., Silipo G., Javitt D.C. (2010). Sensory deficits and distributed hierarchical dysfunction in schizophrenia. Am. J. Psychiatry.

[bb9045] Litvak V., Friston K. (2008). Electromagnetic source reconstruction for group studies. NeuroImage.

[bb0095] Maunsell J.H., van Essen D.C. (1983). The connections of the middle temporal visual area (MT) and their relationship to a cortical hierarchy in the macaque monkey. J. Neurosci. off. J. Neuroimmune. Pharmacol..

[bb0100] Ohzawa I., Sclar G., Freeman R.D. (1982). Contrast gain control in the cat visual cortex. Nature.

[bb0105] Peterson B.S., Skudlarski P., Gatenby J.C., Zhang H., Anderson A.W., Gore J.C. (1999). An fMRI study of Stroop word-color interference: evidence for cingulate subregions subserving multiple distributed attentional systems. Biol. Psychiatry.

[bb0110] Podzebenko K., Egan G.F., Watson J.D.G. (2005). Real and imaginary rotary motion processing: functional parcellation of the human parietal lobe revealed by fMRI. J. Cogn. Neurosci..

[bb9060] Polat U., Norcia A.M. (1996). Neurophysiological evidence for contrast dependent long-range facilitation and suppression in the human visual cortex. Vis. Res..

[bb0115] Purves D., Shimpi A., Lotto R.B. (1999). An empirical explanation of the cornsweet effect. J. Neurosci. off. J. Neuroimmune. Pharmacol..

[bb0120] Purves D., Williams S.M., Nundy S., Lotto R.B. (2004). Perceiving the intensity of light. Psychol. Rev..

[bb0125] Rahnev D., Lau H., de Lange F.P. (2011). Prior expectation modulates the interaction between sensory and prefrontal regions in the human brain. J. Neurosci. off. J. Neuroimmune. Pharmacol..

[bb0130] Rahnev D., Maniscalco B., Graves T., Huang E., de Lange F.P., Lau H. (2011). Attention induces conservative subjective biases in visual perception. Nat. Neurosci..

[bb0135] Rao R.P.N., Ballard D.H. (1999). Predictive coding in the visual cortex: a functional interpretation of some extra-classical receptive-field effects. Nat. Neurosci..

[bb0140] Rauss K., Schwartz S., Pourtois G. (2011). Top-down effects on early visual processing in humans: A predictive coding framework. Neurosci. Biobehav. Rev..

[bb9070] Reynolds J.H., Heeger D.J. (2009). The normalization model of attention. Neuron.

[bb0145] Shikata E., Hamzei F., Glauche V., Koch M., Weiller C., Binkofski F., Büchel C. (2003). Functional properties and interaction of the anterior and posterior intraparietal areas in humans. Eur. J. Neurosci..

[bb0150] Skottun B.C., Skoyles J.R. (2007). Contrast sensitivity and magnocellular functioning in schizophrenia. Vis. Res..

[bb0155] Slaghuis W.L. (1998). Contrast sensitivity for stationary and drifting spatial frequency gratings in positive- and negative-symptom schizophrenia. J. Abnorm. Psychol..

[bb0160] Smith A.T., Baker-Short C.M. (1993). Pharmacological separation of mechanisms contributing to human contrast sensitivity. Vis. Neurosci..

[bb0165] Spratling M.W. (2008). Reconciling predictive coding and biased competition models of cortical function. Front. Comput. Neurosci..

[bb9065] Strelnikov K. (2007). Can mismatch negativity be linked to synaptic processes? A glutamatergic approach to deviance detection. Brain Cogn..

[bb0170] Summerfield C., Trittschuh E.H., Monti J.M., Mesulam M.-M., Egner T. (2008). Neural repetition suppression reflects fulfilled perceptual expectations. Nat. Neurosci..

[bb0175] Weber E., Wagner R. (1846). Der Tatsinn und das Gemeingefuhl. *Handwörterbuch der Physiologie*.

[bb0180] Wyart V., Nobre A.C., Summerfield C. (2012). Dissociable prior influences of signal probability and relevance on visual contrast sensitivity. Proc. Natl Acad. Sci. USA.

